# Landscape pattern has greater influence than local vegetation on songbirds along rights-of-way in forest-dominated landscapes

**DOI:** 10.1371/journal.pone.0351748

**Published:** 2026-07-15

**Authors:** Eric L. Margenau, Petra B. Wood, Christopher W. Ryan

**Affiliations:** 1 West Virginia Cooperative Fish and Wildlife Unit, School of Natural Resources, West Virginia University, Morgantown, West Virginia, United States of America; 2 West Virginia Division of Natural Resources c/o West Virginia University, Morgantown, West Virginia, United States of America; National Museums of Kenya, KENYA

## Abstract

The proliferation of linear energy infrastructure (e.g., pipelines, powerlines) in forested landscapes throughout North America has advanced the need to understand how to manage these areas for improved species conservation. Habitat management implemented to use the unique vegetative characteristics associated with energy corridors may be a promising direction for future conservation. For songbirds, responses to habitat change can vary based on vegetative characteristics at local and landscape scales. Therefore, a better understanding of how vegetative characteristics across spatial scales affect songbird responses to habitat management that is associated with linear energy infrastructure is necessary for developing proactive solutions. We assessed songbird abundance and richness at point count locations within silviculture treatments along energy corridors (pipelines and powerlines) to habitat characteristics at local (within 100 meters surrounding point counts) and landscape (within 500 meters) scales in West Virginia, USA during 2017–2019. Landscape variables had greater influence on songbirds in silviculture treatments compared to local variables. Species- and community-level responses to variables varied depending on habitat guild association, with some within-guild species responding to the same variable in opposing directions. The proportion of young forest habitat and the size of the nearest young forest patch surrounding silviculture treatments had the strongest influences on songbirds of all landscape variables considered. The width of the right-of-way adjacent to silviculture treatments had the strongest influence on songbirds of all local variables considered. These results suggest that consideration of the surrounding landscape is important when planning habitat management in forested areas in association with linear energy infrastructure.

## Introduction

The proliferation of linear energy infrastructure (e.g., utility powerlines, oil/gas pipelines, seismic lines; hereafter rights-of-way, ROWs) has spurred interest in understanding how habitat management strategies can include these areas for creating or enhancing landscapes for diverse songbird communities [1–3]. In forested regions of eastern North America, the presence of energy infrastructure can have profound effects on the songbird community. Pipelines and powerlines that are nested within forested landscapes reduce and fragment forest cover which negatively affects mature forest and interior forest songbirds [4,5]. At the same time, the early-successional vegetation of energy corridors due to periodic maintenance via mowing or herbicide spraying can provide vegetation characteristics suited to early-successional or young forest songbirds. Indeed, management of energy corridors in eastern North America and the central Appalachian region is being used at a greater frequency as a conservation tool to increase the amount of habitat for early-successional and young forest species [6–8]. However, little is known about how vegetation features surrounding energy corridors effects conservation efforts of these areas for songbirds. A better understanding of how surrounding vegetation features influence songbird responses to localized habitat management can help guide decisions and strategies aimed at promoting a diverse songbird community within these landscapes.

Managing songbird habitat in landscapes with ROWs offers unique challenges. Early-successional or young forest vegetation within ROWs is often isolated within predominantly mature forested areas, and the narrow but continuous nature of ROW corridors can span long distances. Isolation of early-successional or young forest vegetation likely affects how effective local scale habitat management can be for associated species [9,10]. Further, vegetation type and associated species composition (e.g., herbaceous material in early-successional areas or woody plant material in young forest areas) in energy corridors likely affects how species respond to management measures. While energy corridors do provide habitat for early-successional and young forest species, active management adjacent to ROWs aimed at improving these areas and optimizing conservation measures has been generally unexplored [11]. The current lack of information on this subject highlights the need for effective management strategies of early-successional and young forest habitats surrounding ROWs. Doing so will enable managers to maximize the suitability of areas near ROWs for songbirds.

Songbirds are highly mobile and use different cover types during the breeding and post-breeding periods to meet their reproductive and dietary needs. Habitat management aimed at enhancing songbird habitats manipulates the local structure and spatial arrangement of vegetation with the intention of creating vegetation characteristics that best align with species’ life history needs. These management strategies often occur at the stand- or local scale because this scale has the most profound effect on species’ habitat requirements [12–14]. However, these strategies can likely be enhanced when considered with larger spatial scales, but management decisions at the local scale requires knowledge of how species respond to vegetation features across multiple spatial scales [15–17]. Despite studies examining how vegetation characteristics across spatial scales affect songbirds within the context of linear energy infrastructure (e.g., [4,18,19]), more study is needed to further develop guidance for how to incorporate local, ROW-focused habitat management measures within these landscapes. Given the isolation of early-successional or young forest vegetations in energy corridors within forested landscapes, the suitability of these habitats for species is likely influenced by the availability of neighboring suitable habitat patches [20,21]. However, which characteristics and to what degree they influence songbird response to local habitat management in ROW landscapes remains unclear. With global energy consumption expected to increase by 70% between 2018 and 2050 [22], there is a growing interest in better understanding how local and landscape vegetation patterns can be incorporated into management plans for lands with energy infrastructure footprints.

We quantified songbird abundance and community species richness in managed forest lands (i.e., cut-back borders) adjacent to rights-of-way to assess the relative importance of local and landscape scale vegetation variables surrounding cut-back borders. The goal of our study was to determine how vegetation variables at different spatial scales affect the response of songbird abundance and richness to tree cutting treatments along right-of-way features within forest dominated landscapes. This study builds off Margenau et al. [11], which assessed songbird response to different cut-back border treatments (harvest size and harvest intensity) but did not examine how the surrounding local and landscape vegetation influenced songbirds in cut-back borders. Specific study objectives were to 1) determine whether local or landscape vegetation features had the strongest influence on young forest-associated songbirds and communities; and 2) determine which local or landscape vegetation features were most important for maintaining mature forest-associated songbirds and communities in these landscapes. We predicted young forest-associated songbirds will respond positively to greater amounts of young forest or early-successional vegetation within the landscape and when young forest patches are closer and larger to surrounding cut-back borders due to the low levels of this cover type and isolation within the predominantly mature forested region. We predicted that mature forest-associated songbirds will respond positively to greater amounts of mature forest and core mature forest vegetation surrounding cut-back borders due to this cover type providing important habitat in areas with linear energy infrastructure. The increased prevalence of linear energy infrastructure has simultaneously created opportunities and challenges for managing diverse songbird communities. Therefore, information on how local vegetation characteristics and landscape scale patterns in land cover affect habitat suitability for songbirds can provide additional guidance for land managers when developing species conservation strategies.

## Methods

### Study area

**Fig 1:**
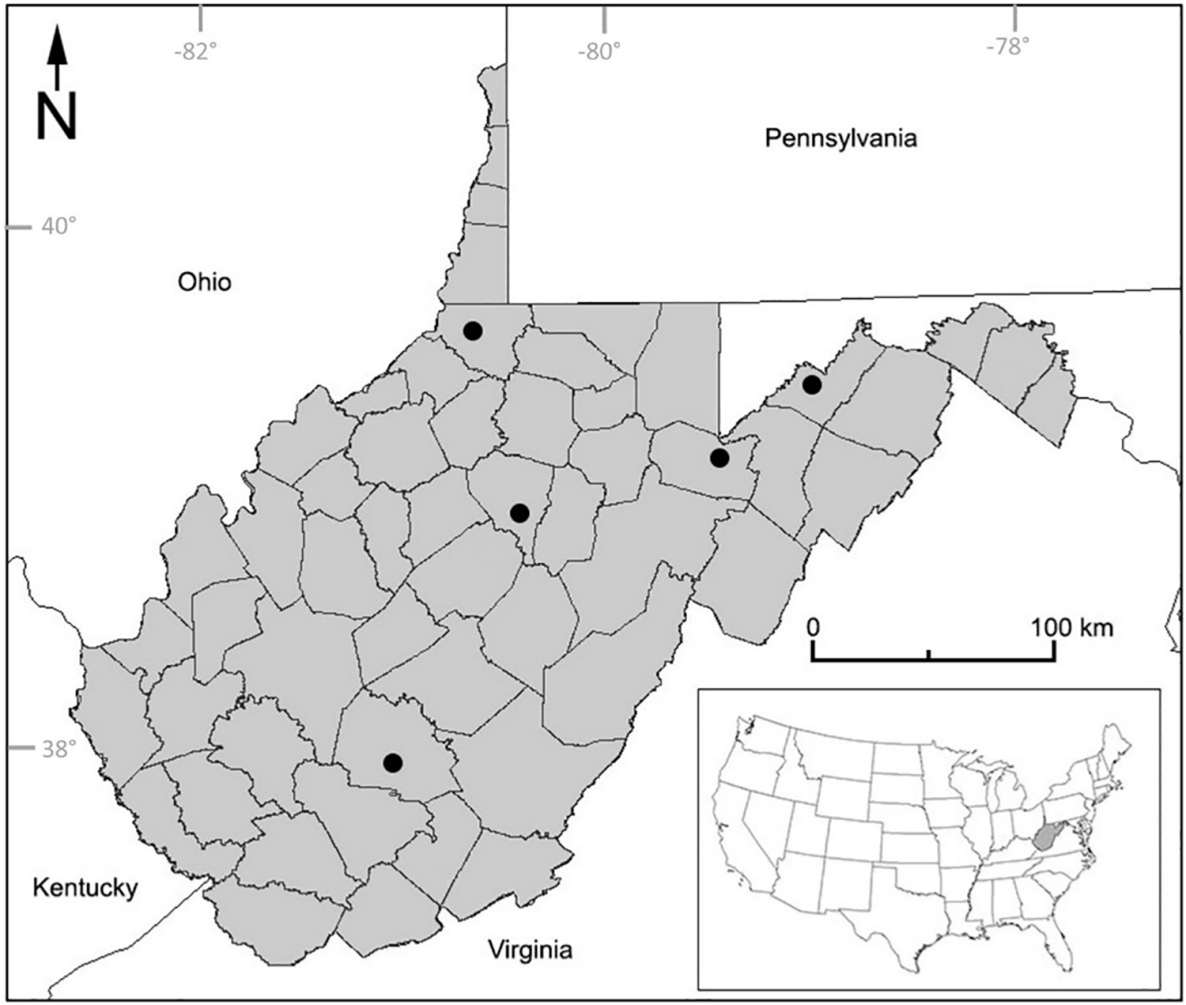
Map of study area. County-level map of West Virginia, USA, showing five study sites (black dots) used to assess songbird response to cut-back borders along pipelines and powerlines during 2017–2019. Map was created using County Boundaries layer from WV GIS Technical Center in ArcGIS v. 10.6 [23].

This study was conducted on five West Virginia Division of Natural Resources’ (WVDNR) wildlife management areas (WMA) located throughout West Virginia, USA (Fig 1). West Virginia lies within the central Appalachian region which is characterized by extensive areas of mature deciduous forest and rugged topography with elevations at study sites ranging from 224 to 1132 m. West Virginia’s humid continental climate is characterized by warm summers (daily mean = 21 °C) and cool to cold winters (daily mean = 1 °C) with mean annual precipitation levels 760, 1270, and 1020 mm in the western, central, and eastern portions of the state, respectively, with heaviest precipitation occurring during spring and summer [24]. Powerline and pipeline ROWs used in this study were narrow linear openings that ranged from 9 to 39 m wide (mean = 25.7, SD = 17.9 m). Vegetation in ROWs consisted of grassland or low shrubs, was void of a mid- or over-story vegetation layer, and was distinct from adjacent forests.

### Experimental design

**Fig 2:**
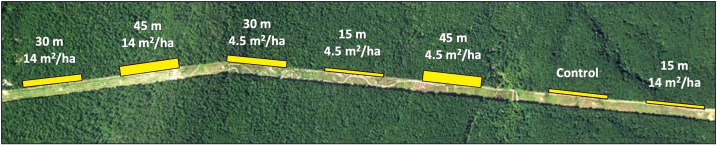
Aerial view of block replicate. Aerial photograph of a cut-back border block replicate along an overhead utility powerline. Cut-back border plots (shown in yellow) were located in mature forests adjacent to right-of-way. Plots were randomly assigned one of two target residual basal area levels (4.5 or 14.0 m^2-ha^ basal area retention) and one of three cutting depths into the forest (15, 30, or 45 m perpendicular to forest edge) with one cut-back border designated as a control (i.e., no tree cutting).

**Fig 3:**
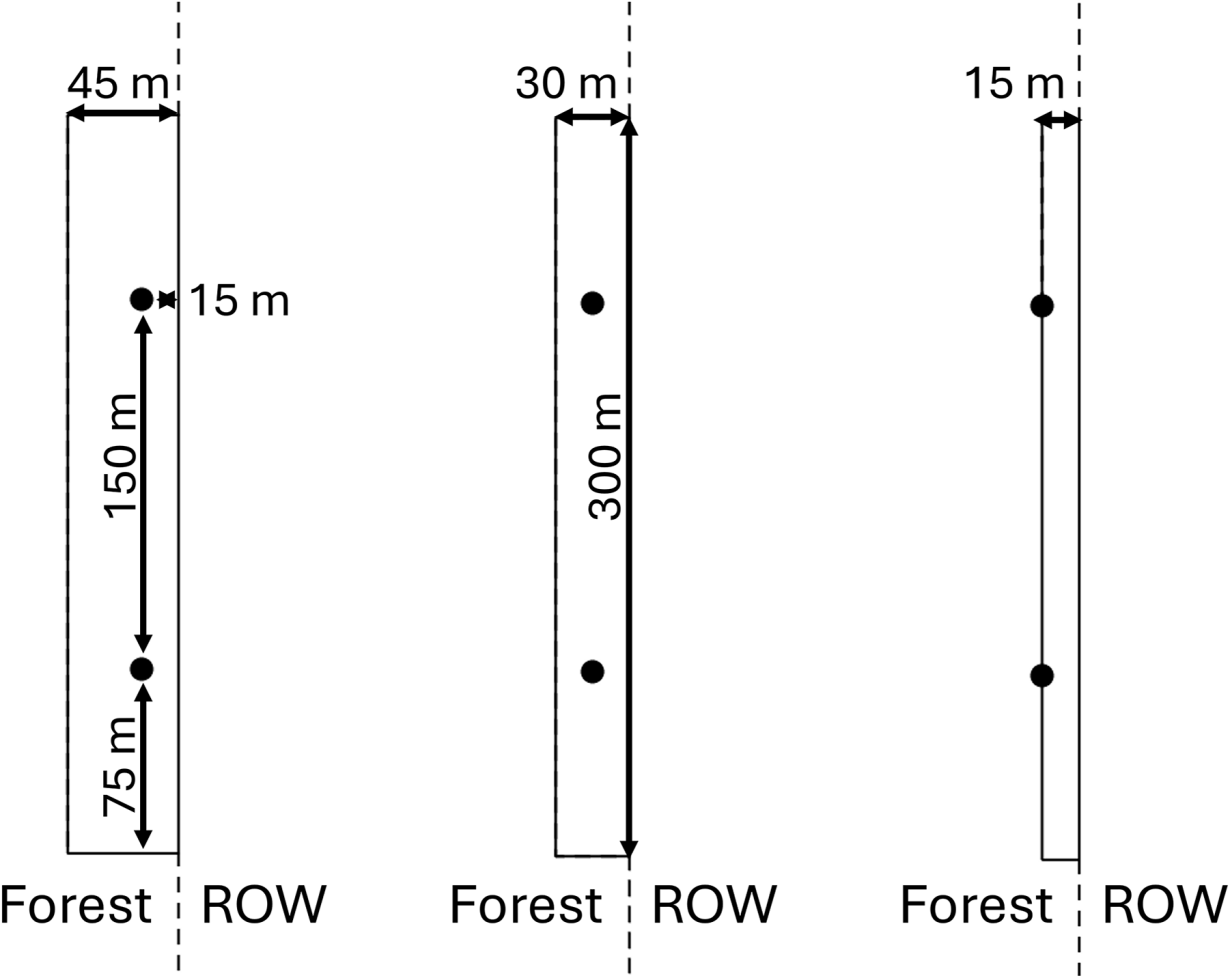
Sampling plot layout. Cut-back border plot layout with avian and vegetation sampling points (black dots) for each harvest width factor level (15, 30, and 45 m) along pipelines and powerlines (“ROW”). Sampling points were located 15 m from forest–ROW boundary (dashed-line) for all harvest width factor levels, 150 m from intraplot sampling points, and 75 m from the plot ends. Cut-back border plot extends 300 m along the forest–ROW boundary.

The eight WMAs included eight block replicates located in mature forests along edges of underground gas pipelines (*n* = 4) or overhead utility powerlines (*n* = 4). A single block replicate consisted of seven independent experimental units (hereafter “cut-back borders”) with each assigned one of seven experimental treatments. We assigned treatments for each block replicate by randomly selecting one of two target residual basal area levels (4.5 m^2-ha^ or 14.0 m^2-ha^ basal area retention to mimic clearcut with reserves and crop tree/shelterwood prescriptions, respectively) and one of three cutting depths into the forest (15 m, 30 m, or 45 m perpendicular to forest edge) for each cut-back border with one cut-back border randomly designated as a control (i.e., no tree cutting in mature forest along the ROWs; Fig 2). Harvest intensities and cutting depths were based on typical prescriptions that WVDNR uses on WMAs. Target basal area in each cut-back border was achieved via tree cutting using a mechanical tree harvester by feller-buncher or by hand crew. Felled trees were dropped within the cut-back border plot and remained on the ground following cutting. Each cut-back border was 300 m in linear distance along the forest edge (300 m × 15 m wide treatment = 0.5 ha total area, 30-m wide treatment = 0.9 ha, and 45-m wide treatment = 1.4 ha) and ≥200 m apart to ensure sampling independence (Fig 3). In each cut-back border, two sampling points were spaced 150 m apart and 15 m from the ROW edge into the forest and were consistent across all cut-back border widths and harvest intensities (Fig 3). These distances for cut-back borders and sampling points were determined based on efficient use of ROWs on WMAs and logistical operability of harvesting equipment. Sampling point location allowed us to sample a large enough area at each point count to encompass a gradient from the ROW, across the cut-back border, and into the adjacent mature forest to evaluate avian responses to cut-back borders. Avian and vegetation data were collected at the same sampling locations at one-year and two-year post-treatment periods.

### Avian sampling

We assessed songbirds in cut-back borders during the summer breeding period using 10-minute standardized fixed-radius point counts [25]. At each point count location, we conducted two annual visits between 18 May–27 June during 2017–2019 to capture peak breeding and singing behavior. Surveys began after 0530 and before 1000 (EST) on days that were considered optimal for sampling (i.e., no rain, no heavy or gusty winds, and minimal peripheral noise). Observers recorded species of each individual bird detected, type of detection (song, call, visual, or flyover), demographic information (male, female, juvenile, or unknown), and distance from observer placed into one of two distance intervals (0–50 m, > 50 m). Survey-level data for each visit included time of survey, ordinal day, and surveyor skill level. Prior to surveys, observers were trained to independently identify all species visually and aurally and estimate distance to the individual while simultaneously recording the distance interval. Surveyor skill level was assessed based on previous experience conducting point counts and identification of avian species of the region and ranged from 3–5. A surveyor score of 5 indicated thorough knowledge of species with extensive experience conducting point counts and a score of 3 indicated introductory knowledge of species with minimal experience conducting point counts. In total, 13 individuals conducted surveys over the four years. For data analyses, we used only adult detections within 50-m of the sampling point and included birds detected by sight and sound (excluding flyovers) for individual species and community statistical analyses. For community analyses, we excluded all species that are not sampled well using point counts (e.g., corvids, raptors, gallinaceous birds; [26]). Sampling permits were not required for point counts.

### Local and landscape scale habitat metrics

We derived local scale variables using vegetation surveys and landscape scale variables using 1-m resolution leaf-on aerial imagery from 2018 National Agriculture Imagery Program [27] and 3-m resolution digital elevation models (DEMs) from West Virginia Statewide Addressing and Mapping Board [28,29], as well as personal knowledge of the study sites. We manually digitized land cover classes (Table 1) in the program ArcGIS v. 10.6 [23] then converted maps to 1-m resolution raster grids. Breaks in land classes (e.g., roads through mature forest) were classed as breaks when they were >3 m in width. Using raster grids, we calculated proportion of land cover for each local and landscape scale land class and core forest area in FRAGSTATS version 4 [30].

**Table 1:** Local- and landscape-level parameter metrics.

Parameter	Mean	Standard deviation	Minimum	Maximum
Local – 100 m radius				
Maintained vegetation (%)	4.9	7.3	0.0	31.4
Width of right-of-way (m)	22.3	11.8	5.0	62.0
Young forest vegetation (%)	25.5	13.6	0.0	56.1
Landscape – 500 m radius				
Core forest (%)	28.5	8.1	6.8	42.7
Distance to nearest young forest patch (m)	166.1	192.9	15.0	965.0
Mature forest (%)	90.2	5.9	64.2	97.2
Size of nearest young forest patch (ha)	4.6	4.7	0.5	15.2
Young forest vegetation (%)	4.1	3.6	0.0	20.6

Local-level (100 m radius) and landscape-level (500 m radius) variables associated with point counts at West Virginia Division of Natural Resources’ Wildlife Management Areas. Width of right-of-way was measured from aerial imagery (NAIP 2018). All other variables were estimated in FRAGSTATS (version 4; McGarigal et al. 2012).

Local scale variables represented data collected at each point-count location or within a 100 m radius. A 100 m radius encompassed a 3.1 ha and represented the local extent surrounding each point count location and roughly approximates the size of a songbird territory [31–33]. Within the 100 m radius surrounding each point-count location, we measured the proportion of young forest (even-aged timber harvest occurring within the past 10 years which included the cut-back border plots) or shrubland vegetation (areas with dense woody understory growth and no overstory cover), proportion of maintained vegetation (vegetations subjected to period maintenance, including grassland, hay field, row crop, or agriculture field), width of ROW corridor, elevation, and topographic relative moisture index. Width of ROW corridor adjacent to each point count location was measured from NAIP aerial photos. Elevation data were derived from DEMs. Topographic relative moisture index was derived from slope percent, slope aspect, slope position, and slope configuration data in DEMs and represents a moisture gradient from xeric to mesic soils [34]. We calculated continuous variables elevation and topographic relative moisture index using zonal statistics in the program ArcGIS [23] to generate a mean value within a 50-m radius surrounding each point count location.

Landscape scale variables represented data within a 500 m radius surrounding each point-count location, which encompassed a 78.5 ha. Previous studies have shown that the 500 m extent captures species responses to landscape-level variables in ROW landscapes [19]. Landscape scale variables included proportion of mature forest (areas where the majority of woody stems are ≥ 28.0 cm DBH), proportion of core forest (defined as mature forest ≥100 m from any edge), proportion of young forest or shrubland vegetation (which included cut-back borders; hereafter “young forest”), distance to nearest young forest patch, and size of nearest young forest patch. The adjacent ROW was excluded from measurement for distance to nearest young forest patch and size of nearest young forest patch variables, as these areas are not considered young forest. All variables were selected based on low collinearity with other variables, assumed importance to the songbird community, ease of measurement and conceptualization, and ability to implement by land managers.

### Statistical analyses

We evaluated the effects of local and landscape scale variables on individual songbird species and avian guilds using stacked Bayesian *N*-mixture models [35]. For individual songbird species analysis, we included species that had ≥ 30 detections and ≥0.2 cumulative detection probability (probability of detecting a species at least once across two annual survey visits). For avian guild richness analysis, we included all species detected within the young forest habitat guild (18 total species detected), forest gap specialist guild (seven species), forest interior habitat guild (19 species), and species of conservation priority (27 species). Species were assigned to guilds based on previous guild studies from the region [19,36]. We considered a species as conservation priority if it was listed by Appalachian Mountains Joint Venture [37], Partners-in-Flight Appalachian region – Bird Conservation Region 28 [38], or WVDNR State Wildlife Action Plan [39].

We fit two candidate models for each species or guild, the first included only local scale vegetation measures (model 1) and the second included only landscape scale vegetation measures (model 2). In addition to local or landscape vegetation variables in the models, we included cut-back border width (0 m [control], 15 m, 30 m, or 45 m) and harvest intensity (no harvest [control], 4.5 m^2-ha^, or 14.0 m^2-ha^ basal area retention) as factors in both models to account for differences in experimental treatments among cut-back borders. Each model was composed of a detection sub-model and an abundance/richness sub-model. We modeled conditional detection probability from two annual survey visits (assuming a closed population within each year and open population across years) with a binomial process model using ordinal day, time since sunrise, and surveyor skill as covariates on the logit-linear scale. No random effects were included in the detection sub-model. For individual songbird analyses, we used the count of unique individuals during each visit as our response variable in our abundance sub-model. For avian guild analyses, we used the number of unique species during each visit as our response variable in our richness sub-model. We modeled expected species abundance or guild richness using a Poisson process model. In both the local and landscape scale models, we included a point count random effect to account for heterogeneity among points and repeated observation at the same point and a treatment plot random effect to account for the lack of independence between points within the same cut-back border. We also included elevation and topographic relative moisture index as nuisance variables to account for topographic variability in local and landscape scale models. All continuous variables were standardized to have a mean of 0 and standard deviation of one prior to analysis. Variables were tested for collinearity prior to analysis.


$$\begin{array}{c} \text{log}(\lambda_{ik})\;=\;\beta \;+\;point\;count\;random\;effect\;+\;treatment\;plot\;random\;effect \end{array}$$

$$\begin{array}{c} +\;elevation\;+\;topographic\;relative\;moisture\;index \end{array}$$

$$\begin{array}{c} +\;cut-back\;border\;harvest\;width \end{array}$$

$$\begin{array}{c} +\;cut-back\;border\;harvest\;intensity \end{array}$$

$$\begin{array}{c} +\;maintained\;vegetation\;within\;\text{1}\text{0}\text{0}\;m \end{array}$$

$$\begin{array}{c} +\;young\;forest\;vegetation\;within\;\text{1}\text{0}\text{0}\;m \end{array}$$

$$\begin{array}{c} +\;width\;of\;ROW\;corridor. \end{array}$$ (1)

$$\begin{array}{c} \text{log}(\lambda_{ik})\;=\;\beta \;+\;point\;count\;random\;effect\;+\;treatment\;plot\;random\;effect \end{array}$$

$$\begin{array}{c} +\;elevation\;+\;topographic\;relative\;moisture\;index \end{array}$$

$$\begin{array}{c} +\;cut-back\;border\;harvest\;width \end{array}$$

$$\begin{array}{c} +\;cut-back\;border\;harvest\;intensity \end{array}$$

$$\begin{array}{c} +\;young\;forest\;vegetation\;within\;\text{5}\text{0}\text{0}\;m \end{array}$$

$$\begin{array}{c} +\;core\;forest\;within\;\text{5}\text{0}\text{0}\;m \end{array}$$

$$\begin{array}{c} +\;mature\;forest\;within\;\text{5}\text{0}\text{0}\;m \end{array}$$

$$\begin{array}{c} +\;distance\;to\;nearest\;young\;forest\;patch \end{array}$$

$$\begin{array}{c} +\;size\;of\;nearest\;young\;forest\;patch \end{array}$$ (2)

We used diffuse prior distributions for all slope coefficients (Gaussian [mean = 0, variance = 10]) because we were uncertain of the relationships between predictor variables and songbirds. For our random point count and treatment plot effects, we used a Gaussian distribution (mean = 0, variance = τ) prior with a hyperparameter τ from an inverse Gamma distribution (α = 1, β = 1).

We discriminated between the local and landscape models for each species or guild using Watanabe-Akaike Information Criterion (WAIC) as our measure of model fit. WAIC was used for model selection due to it being fully a Bayesian measure of model performance and for its application in hierarchical and mixture models [40]. From our best-fit model for each species or guild, we obtained posterior distributions of model parameters by running three parallel Markov chain Monte Carlo (MCMC) simulations of 150000 iterations with a burn-in of 20000 iterations at a thinning rate of 50, yielding 7800 samples for posterior distributions. We considered a variable to have an influential effect on abundance or richness of a given species or guild when the 95% credible interval (CI) around the estimated coefficient did not overlap zero. We assessed model fit with a posterior predictive check, where we compared Chi-square goodness-of-fit tests between an observed posterior predictive distribution and a simulated posterior predictive distribution. We calculated a Bayesian *p*-value, *p*_*B*_, as the probability to obtain a Chi-square test statistic that is at least as extreme as the observed Chi-square test statistic and assumed reasonable fit if 0.1 < *p*_*B*_ < 0.9 [41]. Model convergence was assessed using the Brooks-Gelman-Rubin statistic and assumed adequate convergence when all parameter Ȓ values <1.1 [42]. Data analyses were performed in R [43] using a Bayesian framework in JAGS [44] called from the package jagsUI [45]. We present results as effect sizes, with percent increase or decrease of abundance or richness under a one standard deviation change in the predictor variable. We calculated effect size using the inverse link function on the intercept (*β* in Eqs 1 and 2) and percent increase or decrease represented by a 1-unit change in the variable (i.e., one standard deviation), with all other variables being held at their means. If there was no clear best model between candidate models (i.e., ΔWAIC <2) then we present results from both models.

## Results

Landscape-level land cover within 500 m radius varied across study sites but all were dominated by mature forest cover (mean = 90%) with an average of 29% of this being core forest area (Table 1). Young forest vegetation was the predominant early seral vegetation type at the local-level (100 m radius) with 26% of all land cover (Table 1). Maintained early-successional vegetation comprised 5% of all land cover within 100 m of point counts (Table 1).

In total, nine species from the young forest habitat guild, forest gap specialist habitat guild, forest interior habitat guild, or species of conservation priority met the detection criterion for species specific analyses (Table 2). Our posterior predictive checks indicated that the top selected model for each individual species and avian guild was a reasonable fit (Table 3). Individual species from the young forest habitat guild included three species (common yellowthroat, eastern towhee, and indigo bunting; scientific names for all species are provided in Table 2), the forest gap specialist guild included two species (black-throated green warbler and hooded warbler), the forest interior habitat guild included three species (black-and-white warbler, ovenbird, and wood thrush), and species of conservation priority guild included four species (eastern wood-pewee [also includes eastern towhee, hooded warbler, and wood thrush]).

**Table 2:** Songbird species used in analyses.

Common name	Scientific name	Habitat guild	Count	*p*
Black-and-white Warbler	*Mniotilta varia*	Forest interior	96	0.28
Black-throated Green Warbler	*Setophaga virens*	Forest gap	32	0.31
Common Yellowthroat	*Geothlypis trichas*	Young forest	50	0.34
Eastern Towhee*	*Pipilo erythrophthalmus*	Young forest	128	0.23
Eastern Wood-pewee*	*Contopus virens*	NA	40	0.36
Hooded Warbler*	*Setophaga citrina*	Forest gap	112	0.28
Indigo Bunting	*Passerina cyanea*	Young forest	115	0.25
Ovenbird	*Seiurus aurocapilla*	Forest interior	115	0.27
Wood Thrush*	*Hylocichla mustelina*	Forest interior	38	0.24

Count (total number of detections over all study years), cumulative detection probability (*p*), and habitat guild association of focal species used to assess the influence of local and landscape scale vegetation variables on songbirds in cut-back borders in West Virginia during 2017–2019.

* Species of conservation priority identified by Appalachian Mountains Joint Venture (AMJV 2018), Partners-in-Flight Appalachian region – Bird Conservation Region 28 (PIF 2019), or WVDNR State Wildlife Action Plan (WVDNR 2015).

**Table 3:** Model results for songbird species and community guilds.

Species	Model scale	WAIC	ΔWAIC	χ^2^
Black-and-white Warbler	Local	3926.6	2.7	0.26
	Landscape	3923.9	–	0.25
Black-throated Green Warbler	Local	3704.3	–	0.27
	Landscape	3712.5	8.2	0.26
Common Yellowthroat	Local	3757.6	10.1	0.41
	Landscape	3747.5	–	0.39
Eastern Towhee	Local	4132.3	–	0.25
	Landscape	4132.6	0.3	0.21
Eastern Wood-pewee	Local	3757.6	16.6	0.39
	Landscape	3741.0	–	0.34
Hooded Warbler	Local	3972.8	–	0.41
	Landscape	3976.2	3.4	0.41
Indigo Bunting	Local	3986.7	–	0.48
	Landscape	3988.4	1.7	0.42
Ovenbird	Local	3969.9	–	0.39
	Landscape	3976.3	6.4	0.40
Wood Thrush	Local	3729.7	1.9	0.21
	Landscape	3727.8	–	0.20
**Guild associations**				
Young Forest habitat	Local	4617.4	1.4	0.51
	Landscape	4616.0	–	0.50
Forest Gap Specialist habitat	Local	4459.7	0.6	0.54
	Landscape	4459.1	–	0.52
Forest Interior habitat	Local	4634.9	2.1	0.42
	Landscape	4632.8	–	0.43
Species of Conservation Priority	Local	4823.7	–	0.69
	Landscape	4829.1	5.4	0.69

Watanabe-Akaike Information Criterion (WAIC) scores, ΔWAIC and Chi-square goodness-of-fit test (χ^2^) of local and landscape models for songbird species and guilds.

**Fig 4:**
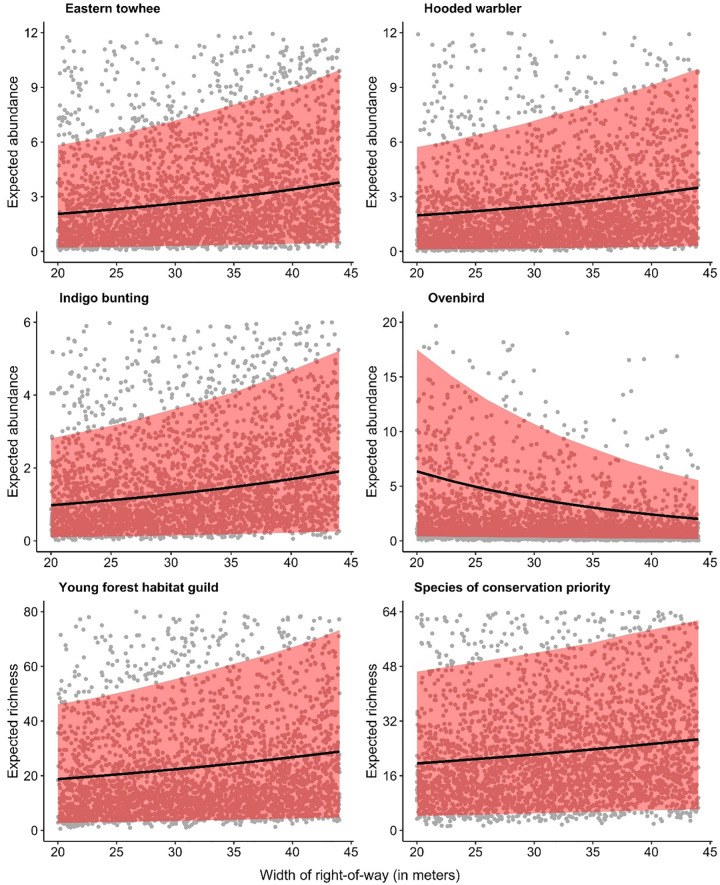
Songbird response to “Width of right-of-way” variable. Expected point-level mean abundance or richness (gray dots) with 85% credible interval of eastern towhee, hooded warbler, indigo bunting, ovenbird, young forest habitat guild, and species of conservation priority guild as a function of right-of-way width in meters. Black line indicates mean expected abundance or richness. Values of “Width of right-of-way (in meters)” on x-axis were truncated at the first and third quartile of their distribution to represent core values.

The local scale model was the top selected model for five species: black-throated green warbler, eastern towhee, hooded warbler, indigo bunting, and ovenbird and the species of conservation priority guild (Table 3). Additionally, the local scale model for wood thrush, young forest guild, and forest gap specialist guild was < 2 ΔWAIC to the landscape scale model (Table 3), indicating no clear best model for these species and thus results are included for these species as well. Abundances of eastern towhee, hooded warbler, and indigo bunting and species richness of the young forest habitat and species of conservation priority guilds were positively influenced by increasing ROW width, with predicted effect size increases of 71.1% (95% Credible Interval: 12.4–160.4%), 74.5% (11.2–184.3%), 77.4% (16.9–178.2%), 42.0% (10.4–82.6%), and 28.0% (3.1–58.6%), respectively, when the ROW increased by 11.8 m (i.e., one standard deviation change). Abundance of ovenbirds was negatively influenced by increasing ROW width, decreasing by a predicted 51.2% (17.4–72.2%) (Fig 4). Abundances of black-throated green warbler and wood thrush and species richness of the forest gap specialist guild in cut-back borders were not strongly influenced by local scale vegetation variables.

**Fig 5:**
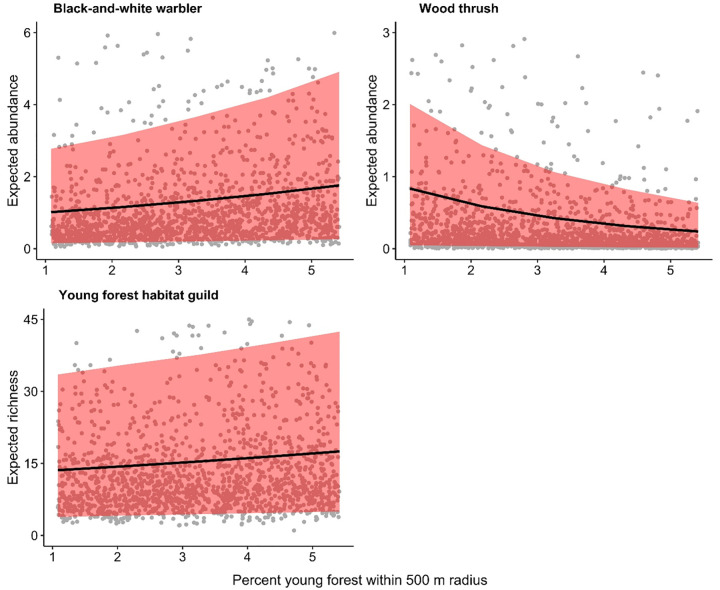
Songbird response to “Percent young forest within 500 m radius” variable. Expected point-level mean abundance or richness (gray dots) and 85% credible interval of black-and-white warbler, wood thrush, and young forest habitat guild as a function of the percentage of young forest vegetation within 500 meters surrounding point counts in cut-back borders. Each paneled figure has a unique y-axis range specific to the mean abundance estimates. Black line indicates mean expected abundance or richness. Values of “Percent young forest within 500 m radius” on x-axis were truncated at the first and third quartile of their distribution to represent core values.

**Fig 6:**
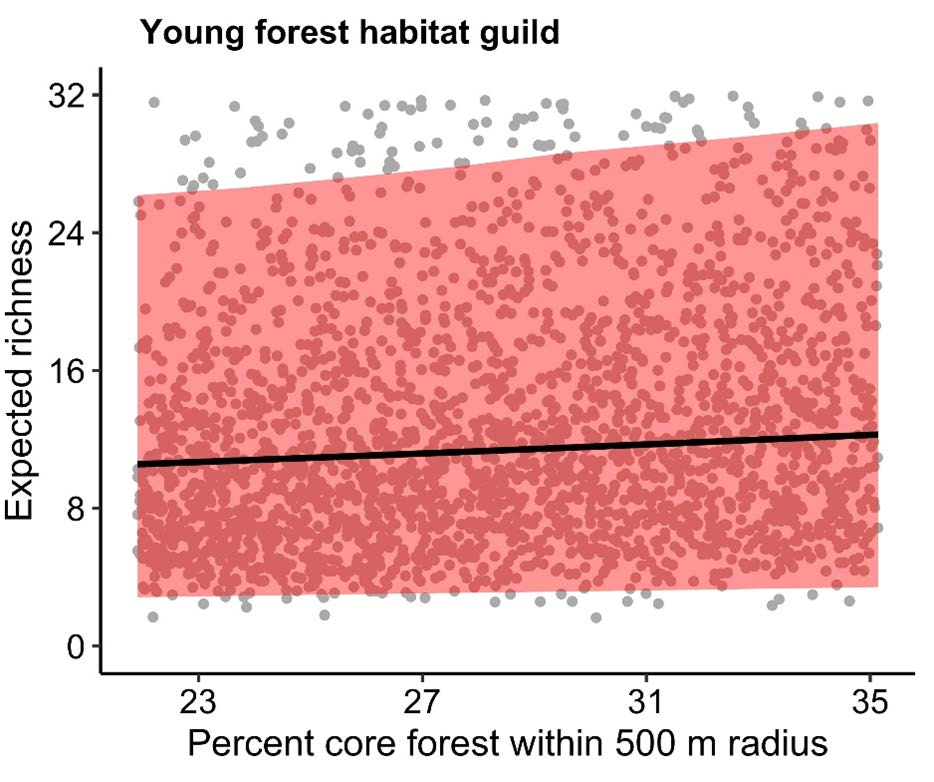
Songbird response to “Percent core forest within 500 m radius” variable. Expected point-level mean richness (gray dots) and 85% credible interval of the young forest habitat guild as a function of percentage core forest vegetation (mature forest ≥100 m from any edge) within 500 m radius surrounding point counts in cut-back borders. Black line indicates mean expected richness. Values of “Percent core forest within 500 m radius” on x-axis were truncated between the first and third quartile of their distribution to represent core values.

**Fig 7:**
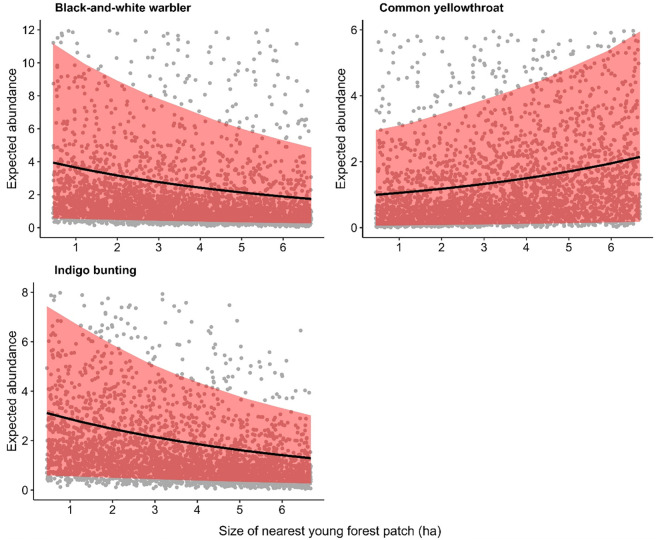
Songbird response to “Size of nearest young forest patch” variable. Expected point-level mean abundance (gray dots) of black-and-white warbler, common yellowthroat, and indigo bunting with 85% credible interval as a function of the size of the nearest young forest patch (in hectares) surrounding point counts in cut-back borders. Each paneled figure has a unique y-axis range specific to the mean abundance estimates. Black line indicates mean expected abundance. Values of “Size of nearest young forest patch (ha)” on x-axis were truncated at the first and third quartile of their distribution to represent core values.

The landscape scale model was the top model for four species: black-and-white warbler, common yellowthroat, eastern wood-pewee, and wood thrush and three guilds: the young forest guild, the forest gap specialist guild, and the forest interior guild (Table 3). Additionally, the landscape scale model for eastern towhee and indigo bunting was < 2 ΔWAIC to the local scale model (Table 3). Abundance of black-and-white warblers and species richness of the young forest guild were positively influenced by increasing amounts of young forest vegetation within 500 m surrounding cut-back borders, with predicted increases of 85.0% (11.7–226.7%) and 32.3% (3.4–70.6%), respectively, when young forest increased by 3.6% (or 2.8 ha) (Fig 5). Abundance of wood thrush was negatively influenced by young forest, with a predicted decrease of 73.7% (7.7–94.2%) (Fig 5). Species richness of the young forest habitat guild was positively influenced by the amount of core forest within 500 m, with a predicted increase of 30.5% (2.4–66.2%) when core forest increased by 8.1% (or 6.4 ha) (Fig 6). Abundance of common yellowthroats was positively influenced by the size of the nearest young forest patch, with a predicted increase of 165.6% (2.5–656.9%) when the nearest young forest patch increased by 4.3 ha, while abundances of black-and-white warbler and indigo bunting were negatively influenced by nearest patch size, with predicted decreases of 58.4% (7.3–83.0%) and 60.8% (21.3–81.8%), respectively (Fig 7). Abundances of eastern towhee and eastern wood-pewee and species richness of the forest gap specialist guild and forest interior guild were not strongly influenced by any vegetation variables at the landscape scale.

## Discussion

The proliferation of linear energy infrastructure in West Virginia, the Appalachian region, and the eastern US has provided an opportunity for land managers to incorporate these man-made features into their management plans to manage for a diverse songbird community. We evaluated the effects of vegetation variables at local and landscape scales on songbirds in cut-back borders along ROW corridors and found that vegetation composition at the landscape scale more strongly influenced songbird responses. Although the amount of young forest within a 500 m radius surrounding cut-back borders only accounted for 4.1% of landcover on average (3.2 of 78.5 ha in the 500 m radius), an increase of 2.8 ha of young forest had a positive influence on black-and-white warblers and the young forest habitat guild. The amount of young forest within the landscape had a strong, positive influence on the forest interior species, black-and-white warbler, and this relationship suggests that within these mature forest-dominated landscapes, small increases in young forest areas can result in increases in habitat suitability of cut-back borders for some forest interior songbirds. This may be due to young forests providing resources that are not available in mature forests. For example, mature forest-associated species use edges of small young forest patches within contiguous mature forests for foraging, perching, and preening activities due to the increased vertical vegetation diversity provided in these areas [46–48]. Young forest patches are also frequently used by mature forest-associated species during the post-breeding period due to the abundance of food resources and vegetative cover [36,49,50]. The apparent benefits of young forests may result in breeding mature forest adults locating nests closer to young forest patches where fledging birds can access these patches during the post-breeding period [51,52]. Therefore, cut-back borders located in areas with greater amounts of young forest may increase juvenile’s access to other young forest areas, and not just young forests in cut-back borders. However, the relationship with young forests was not consistent for all forest interior species, as wood thrush had a negatively relationship with the amount of young forest in the area. Clipp et al. [53] reported lower reproductive success of wood thrush in actively harvested landscapes compared to minimally harvest landscapes in West Virginia, while Schlossberg et al. [54] found no difference in wood thrush reproductive metrics between landscapes with low (1%) or high (20%) cover of early-successional vegetation in Massachusetts. Wood thrush are a species of regional conservation priority thought to be sensitive to forest loss and fragmentation [55,56]. With our study sites being predominantly mature forests, the location of cut-back borders along forest edges may not be ideal areas for wood thrush and additional increases in young forests may further lower habitat suitability of cut-back borders for wood thrush.

Surprisingly, no individual young forest species responded to increased total amount of young forest at the local or landscape scale, although the young forest habitat guild responded positively to the amount of young forest within 500 m surrounding cut-back borders. This latter finding follows our expectation that young forest-associated species would benefit from having more young forest vegetation in a landscape that is predominantly mature forest. Shoe [57] reported that young forest species occurrence was positively associated with shrubland vegetation within a 500 m buffer surrounding point counts in human-modified landscapes (e.g., sand and gravel mines, transmission line rights-of-way), however, Askins et al. [20] reported surrounding landscape composition was not an important predictor of abundance of young forest species in wildlife openings. Studies have shown that young forest species in ROWs are likely limited by the amount of surrounding early-successional vegetation [58,59], and therefore we would expect this variable to strongly influence species in these areas. Higher levels of early-successional or young forest vegetation surrounding other small habitat patches, like wildlife openings, can increase local young forest species abundances [20,21,60]. This trend is similar for ROW landscapes, as Farwell et al. [19] reported early-successional species were strongly influenced by edge density within 100 m (a variable that had a strong, positive correlation with young forest vegetation within a 100 m radius in our study and thus not included in our analysis). Although, our results indicate that the amount of young forest vegetation is not an important driver of individual species’ abundance in cut-back borders, the young forest bird community was influenced by greater amounts of young forest on the landscape.

The young forest bird community also showed a positive relationship with the amount of core mature forest surrounding cut-back borders. Studies have shown that young forest-associated species use mature forests during the post-breeding period [61], however, the apparent benefit of having greater amounts of core mature forest surrounding cut-back borders or other small young forest patches has not been observed. Young forest species are thought to have a greater tolerance for forest edges [19], though they may also benefit from areas with core mature forest conditions (e.g., reduced microclimate variability with wetter, cooler conditions; [62]) in similar ways that mature forest interior species benefit from these habitats. Further study on potential benefits and drivers for young forest species use of core mature forest vegetation and whether species can differentiate between mature forest and core mature forest would be insightful and help guide management in these landscapes.

While young forest-associated species showed no response to the proportion of young forest vegetation, two species (common yellowthroat and indigo bunting) showed relationships to the size of nearby young forest patches. Common yellowthroat was positively influenced by size of the nearest young forest patch while the indigo bunting was negatively influenced by the size of the nearest young forest patch, which is consistent with previous studies on patch size relationships for these species [58,63]. Similar studies have indicated that patch size relationships tend to be species-specific [60,64] which may explain why we did not see a response in the young forest bird community (i.e., too much species-to-species variability for a consistent directional response). Young forest species can occupy small patches of young forest vegetation within predominately forested landscapes (0.1 ha in [65]; 0.23 ha in [21]). Nearby young forest patches in our study ranged 0.45–15.24 ha in size (29% of all patches were ≤1 ha), which may allow young forest species to occupy cut-back borders and adjacent young forest patches within these landscapes. Along with the size of the nearest young forest patch, we expected that the distance to the nearest young forest patch would also influence responses of young forest-associated species. In theory, the proximity to other young forest areas likely enhances cut-back borders by providing greater access to resources that young forest species are proficient at exploiting as well as promoting these areas through conspecific attraction [66,67]. Despite our prediction we observed no strong relationship between species and distance to the nearest young forest patch. Nevertheless, our findings do indicate that having pre-existing young forest patches in landscapes surrounding ROWs, regardless of patch size, enhances the value of cut-back borders for young forest-associated species.

Width of the right-of-way located adjacent to cut-back borders was the only local-scale variable that strongly influenced species responses in cut-back borders, but it also was the single most influential variable that we considered at both spatial scales. Positive relationships with wider ROWs were mostly by young forest species (eastern towhee, indigo bunting, and the young forest habitat guild) but also the species of conservation priority guide, which included seven young forest species of the total 27 species in this guild, as well as the hooded warbler (a forest gap specialist). ROWs are mainly comprised of grasses, shrubs, or other early-successional plant material that young forest species likely use for foraging or cover purposes. Due to periodic mowing or herbicide treatment, ROWs are typically void of saplings or other structural characteristics typical of young forests. Young forest-associated songbirds are often able to use early-successional vegetation (grasslands, shrublands) interchangeably with young forests, and wider ROWs (i.e., more grassland or shrubland) likely offer similar but not completely overlapping resource communities with the young forest vegetation present in cut-back borders. The complementary nature of early-successional vegetation in ROWs to young forests in cut-back borders highlights how increasing vegetation diversity, through forest management, can improve these areas for young forest-associated songbirds. While wider ROWs had a positive influence on young forest species, wider cut-back borders (“cut-back border harvest width” variable in Eqs 1 and 2) did not influence species responses (Appendix A). Margenau et al. [11] found that the narrowest (15-m) and widest (45-m) cut-back border harvest widths had the strongest positive influences on the young forest habitat guild. This varied response likely limited a clear relationship between songbirds and cut-back border width in our study.

## Conclusion

There is limited information on how best to manage the forest bird community using ROW areas within the context of the surrounding landscape. This research helps fill this knowledge gap on what vegetation characteristics and at what spatial scales are important for the management and conservation of forest songbirds. Of the variables that we assessed, our results indicated that landscape-level variables were more influential to species abundance and richness in cut-back borders than local-level variables, although the width of adjacent right-of-way was the single most influential predictor. The positive relationships we observed to wider rights-of-way adjacent to cut-back borders support the notion that installing new pipelines/powerlines adjacent to existing rights-of-way, instead of creating new rights-of-way in core forests, to minimize increasing forest fragmentation. However, our findings do not directly test this notion, nor, to the author’s knowledge, has this been tested through other empirical field studies. Additionally, some of our predictions had wide CIs. Such large uncertainties observed in our predictions should invite caution when translating or extrapolating our findings to other energy corridors and other forest systems. However, consideration of local- and landscape-scale vegetation features and understanding their effects on songbirds is important information for managers when developing and implementing habitat management measures in association with linear energy infrastructure to support a diverse songbird community. Our findings add to the growing body of literature showing that songbirds, regardless of their nesting habitat preferences, require diverse forest age classes to meet their resource needs. Future research on thresholds of landscape-level vegetation for young forest and mature forest songbirds in cut-back borders will help inform managers about the surrounding limitations of vegetation related to cut-back borders in forest-dominated landscapes. Additionally, longer-term monitoring to fully evaluate spatio-temporal effects of local- and landscape-level vegetation on songbirds will be valuable for developing long-term management plans.

## Supporting information

S1 Table Model results. Mean and 95% lower credible interval–95% upper credible interval on the log scale from local scale and landscape scale models for songbird species and guilds. Variables with 95% credible intervals (CI) that do not overlap 0 are in **bold** typeface and indicate they influenced species or guild responses in cut-back borders. Songbird species and guild abbreviations in table are black-and-white warbler (BAWW), black-throated green warbler (BTNW), common yellowthroat (COYE), eastern towhee (EATO), eastern wood-pewee (EAWP), hooded warbler (HOWA), indigo bunting (INBU), ovenbird (OVEN), wood thrush (WOTH), young forest habitat guild (YF), forest gap specialist guild (FG), forest interior habitat guild (FI), and species of conservation priority (SCP), and local scale predictor variable abbreviations include right-of-way (ROW) and topographic relative moisture index (TRMI).
(PDF)
