# Supplementary material for: Landscape pattern has greater influence than local vegetation on songbirds along rights-of-way in forest-dominated landscapes
Source: PLoS One. 2026 Jul 15;21(7):e0351748. doi: 10.1371/journal.pone.0351748 (PMC13372147; doi:10.1371/journal.pone.0351748)
Supplement: S1 Table — Mean and 95% lower credible interval–95% upper credible interval on the log scale from local scale and landscape scale models for songbird species and guilds. Variables with 95% credible intervals (CI) that do not overlap 0 are in bold typeface and indicate they influenced species or guild responses in cut-back borders. Songbird species and guild abbreviations in table are black-and-white warbler (BAWW), black-throated green warbler (BTNW), common yellowthroat (COYE), eastern towhee (EATO), eastern wood-pewee (EAWP), hooded warbler (HOWA), indigo bunting (INBU), ovenbird (OVEN), wood thrush (WOTH), young forest habitat guild (YF), forest gap specialist guild (FG), forest interior habitat guild (FI), and species of conservation priority (SCP), and local scale predictor variable abbreviations include right-of-way (ROW) and topographic relative moisture index (TRMI). (PDF) [file pone.0351748.s001.pdf]

	BAWW	BTNW	COYE	EATO	EAWP	HOWA
**Local scale variables**						
Proportion of young forest vegetation	0.05 (-0.31-0.38)	0.25 (-0.61-0.93)	-0.19 (-1.33-0.71)	-0.25 (-0.77-0.14)	-0.46 (-1.40-0.20)	-0.38 (-1.06-0.12)
Proportion of maintained vegetation	0.16 (-0.67-1.00)	0.24 (-1.09-1.54)	-0.30 (-1.75-1.12)	-0.36 (-1.19-0.33)	-1.00 (-2.26-0.23)	0.12 (-0.79-0.99)
Width of ROW corridor adjacent to cut-back border	0.28 (-0.20-0.74)	0.03 (-0.80-0.84)	0.72 (-0.14-1.59)	**0.54 (0.12-0.96)**	0.34 (-0.38-1.07)	**0.56 (0.11-1.05)**
Cut-back border harvest intensity	0.30 (-0.28-0.95)	0.30 (-0.62-1.20)	-0.45 (-1.55-0.59)	0.34 (-0.10-0.85)	0.44 (-0.40-1.27)	0.24 (-0.32-0.87)
Cut-back border harvest width	-0.04 (-0.87-0.72)	-0.56 (-1.63-0.54)	0.26 (-0.88-1.41)	0.38 (-0.21-1.15)	0.85 (-0.28-1.97)	-0.16 (-0.97-0.65)
TRMI	-0.01 (-0.46-0.43)	0.89 (-0.07-1.92)	0.41 (-0.50-1.38)	0.25 (-0.11-0.61)	0.01 (-0.65-0.64)	-0.07 (-0.49-0.35)
Elevation	-0.47 (-1.11-0.14)	0.71 (-0.27-1.79)	0.36 (-0.67-1.46)	-0.22 (-0.66-0.22)	-0.50 (-1.37-0.32)	**-1.02 (-1.62--0.47)**
**Landscape scale variables**						
Proportion of young forest vegetation	**0.62 (0.11-1.18)**	-0.39 (-1.36-0.47)	0.34 (-0.65-1.41)	0.30 (-0.07-0.67)	-0.20 (-1.16-0.65)	0.38 (-0.11-0.88)
Proportion of core forest vegetation	0.36 (-0.13-0.87)	0.11 (-0.69-0.93)	0.43 (-0.48-1.42)	0.37 (-0.01-0.75)	0.23 (-0.50-1.04)	0.12 (-0.30-0.57)
Proportion of mature forest vegetation	0.26 (-0.39-0.94)	-0.55 (-1.38-0.27)	0.12 (-0.97-1.33)	-0.11 (-0.54-0.34)	-0.21 (-1.01-0.65)	0.06 (-0.51-0.68)
Distance to nearest young forest patch	0.12 (-0.37-0.56)	-0.60 (-2.12-0.66)	0.16 (-0.98-1.24)	0.15 (-0.24-0.54)	0.30 (-0.39-0.98)	-0.24 (-0.71-0.21)
Size of nearest young forest patch	**-0.88 (-1.77--0.08)**	0.03 (-0.85-0.96)	**0.98 (0.03-2.02)**	0.18 (-0.29-0.62)	-0.58 (-1.91-0.64)	-0.12 (-0.81-0.57)
Cut-back border harvest intensity	0.15 (-0.50-0.80)	0.36 (-0.58-1.33)	-0.63 (-1.69-0.33)	0.28 (-0.18-0.79)	0.48 (-0.45-1.38)	0.02 (-0.60-0.61)
Cut-back border harvest width	-0.12 (-0.56-0.31)	-0.45 (-1.17-0.22)	-0.07 (-0.78-0.59)	-0.06 (-0.39-0.24)	0.12 (-0.47-0.74)	-0.23 (-0.65-0.18)
TRMI	0.07 (-0.40-0.54)	0.89 (-0.14-1.96)	0.34 (-0.58-1.36)	0.18 (-0.19-0.56)	0.01 (-0.70-0.67)	-0.15 (-0.60-0.29)
Elevation	-0.27 (-1.06-0.44)	0.68 (-0.44-1.92)	-0.31 (-1.54-0.96)	-0.21 (-0.73-0.33)	-0.03 (-1.06-0.92)	**-0.83 (-1.66--0.12)**

	INBU	OVEN	WOTH	YF	FG	FI
**Local scale variables**						
Proportion of young forest vegetation	-0.25 (-0.79-0.13)	0.01 (-0.44-0.39)	-0.45 (-1.74-0.42)	-0.09 (-0.33-0.13)	-0.19 (-0.47-0.05)	0.00 (-0.22-0.20)
Proportion of maintained vegetation	0.08 (-0.67-0.75)	-0.02 (-0.91-0.94)	0.27 (-1.25-1.88)	0.13 (-0.32-0.56)	-0.05 (-0.55-0.47)	-0.04 (-0.49-0.41)
Width of ROW corridor adjacent to cut-back border	**0.57 (0.16-1.02)**	**-0.72 (-1.28--0.19)**	-0.46 (-1.56-0.45)	**0.35 (0.10-0.60)**	0.19 (-0.09-0.47)	-0.13 (-0.39-0.13)
Cut-back border harvest intensity	0.34 (-0.18-0.91)	-0.43 (-1.10-0.21)	-0.08 (-1.18-1.02)	0.24 (-0.05-0.55)	0.13 (-0.19-0.44)	-0.07 (-0.39-0.26)
Cut-back border harvest width	0.01 (-0.60-0.69)	0.10 (-0.71-0.84)	-0.19 (-1.56-1.05)	-0.12 (-0.51-0.27)	-0.01 (-0.47-0.43)	0.05 (-0.33-0.44)
TRMI	-0.09 (-0.49-0.30)	-0.36 (-0.96-0.20)	0.47 (-0.41-1.34)	0.15 (-0.10-0.39)	0.22 (-0.05-0.48)	0.05 (-0.21-0.29)
Elevation	**-0.90 (-1.49--0.37)**	**0.95 (0.25-1.69)**	-0.80 (-1.95-0.26)	**-0.42 (-0.71--0.13)**	**-0.49 (-0.83--0.17)**	0.04 (-0.27-0.34)
**Landscape scale variables**						
Proportion of young forest vegetation	0.29 (-0.14-0.75)	-0.31 (-1.04-0.35)	**-1.34 (-2.84--0.08)**	**0.28 (0.03-0.53)**	0.18 (-0.10-0.47)	0.02 (-0.26-0.30)
Proportion of core forest vegetation	0.27 (-0.14-0.68)	-0.22 (-0.76-0.32)	-0.05 (-1.01-0.89)	**0.27 (0.02-0.51)**	0.01 (-0.25-0.28)	0.05 (-0.20-0.29)
Proportion of mature forest vegetation	-0.40 (-0.87-0.10)	0.35 (-0.23-0.95)	0.02 (-0.96-1.13)	-0.15 (-0.43-0.12)	0.00 (-0.31-0.32)	0.09 (-0.20-0.38)
Distance to nearest young forest patch	-0.16 (-0.61-0.29)	0.39 (-0.18-0.96)	-0.44 (-1.29-0.37)	0.04 (-0.22-0.29)	-0.01 (-0.30-0.27)	-0.02 (-0.29-0.24)
Size of nearest young forest patch	**-0.94 (-1.71--0.24)**	0.27 (-0.40-0.95)	0.21 (-1.10-1.52)	0.07 (-0.23-0.38)	-0.10 (-0.46-0.25)	-0.23 (-0.57-0.09)
Cut-back border harvest intensity	0.35 (-0.21-0.89)	-0.28 (-0.92-0.35)	-0.04 (-1.10-0.99)	0.19 (-0.13-0.48)	0.11 (-0.24-0.45)	-0.09 (-0.40-0.22)
Cut-back border harvest width	-0.14 (-0.51-0.25)	0.16 (-0.27-0.62)	0.29 (-0.42-0.99)	-0.17 (-0.37-0.04)	-0.11 (-0.35-0.12)	0.05 (-0.16-0.26)
TRMI	-0.09 (-0.51-0.32)	-0.36 (-0.95-0.22)	0.54 (-0.29-1.41)	0.12 (-0.13-0.37)	0.17 (-0.10-0.44)	0.09 (-0.16-0.34)
Elevation	-0.12 (-0.75-0.50)	0.78 (-0.07-1.70)	-0.62 (-1.95-0.54)	-0.31 (-0.65-0.05)	-0.35 (-0.74-0.04)	0.02 (-0.35-0.40)

	SCP
**Local scale variables**	
Proportion of young forest vegetation	-0.13 (-0.33-0.05)
Proportion of maintained vegetation	-0.09 (-0.48-0.30)
Width of ROW corridor adjacent to cut-back border	**0.25 (0.03-0.46)**
Cut-back border harvest intensity	0.20 (-0.05-0.47)
Cut-back border harvest width	0.09 (-0.24-0.44)
TRMI	0.18 (-0.02-0.38)
Elevation	**-0.41 (-0.66--0.16)**
**Landscape scale variables**	
Proportion of young forest vegetation	0.17 (-0.06-0.41)
Proportion of core forest vegetation	0.13 (-0.08-0.34)
Proportion of mature forest vegetation	-0.05 (-0.29-0.20)
Distance to nearest young forest patch	0.02 (-0.20-0.25)
Size of nearest young forest patch	-0.13 (-0.41-0.15)
Cut-back border harvest intensity	0.17 (-0.12-0.48)
Cut-back border harvest width	-0.06 (-0.28-0.14)
TRMI	0.17 (-0.04-0.38)
Elevation	-0.26 (-0.57-0.03)
